# Dust and Disease

**Published:** 1890-03-08

**Authors:** 


### HYGIENE.

### DUST AND DISEASE.

Dr. R. Stern, in devising a course of research into the efficacy of ventilation, sweeping, &c., in the removal of the germs and other vehicles of disease, arranged a special room for the purpose at the Hygienic Institute, at Breslau, with several ingenious apparatus for the diffusion and collection of dust from the air, floors, &c. Dust from ordinary dwelling rooms, from school desks, from a felt hat factory, the rag rooms of paper works, &c., was sterilized and then impregnated with the bacillus megatherium; and the spores of aspergillus niger were added to other dust in like manner. The room having been previously thoroughly cleansed, the dusts were diffused in the air, and on pieces of carpet, &c., by a Petri's sand filter, and at the conclusion of each experiment the dust collected for observation was sown in gelatin with agar-agar.

The chief conclusions at which he arrived were: (1) That in a still room, with closed windows, subsidence alone is sufficient to rid the air of all ponderable dust; (2) That ventilation, involving not more than four or five changes of the atmosphere in the hour impedes subsidence, while in itself quite insufficient to remove the dust; (3) That ventilation renewing the air six to eight times in an hour, which, although analagous to the currents in the contrivances now frequently annexed to grinding lathes and the like is utterly inadmissible in occupied rooms, is required to carry off the dust diffused in the air, and (4) that water vapour though it may aid the subsidence of dust in a still atmosphere is otherwise useless.

A practical lesson for the housekeeper taught by these experiments is that the only way to insure the removal of dust from a room, and not merely its redistribution, is damp wiping of furniture, floors, &c., with the least possible draught or disturbance of the air, after which, especially if the day be windy, the room may be ventilated and dried by throwing open doors and windows.. The ordinary housemaid's work is the Sisyphus-like labour of raising the dust from one place that it may deposit itself later in another, whereas the constant rinsing of the damp cloths bears witness to the mass of dust taken away, not merely dispersed.

